# Serpins in Hemostasis as Therapeutic Targets for Bleeding or Thrombotic Disorders

**DOI:** 10.3389/fcvm.2020.622778

**Published:** 2021-01-07

**Authors:** Elsa P. Bianchini, Claire Auditeau, Mahita Razanakolona, Marc Vasse, Delphine Borgel

**Affiliations:** ^1^HITh, UMR_S1176, Institut National de la Santé et de la Recherche Médicale, Université Paris-Saclay, Le Kremlin-Bicêtre, France; ^2^Service de Biologie Clinique, Hôpital Foch, Suresnes, France; ^3^Laboratoire d'Hématologie Biologique, Hôpital Necker, APHP, Paris, France

**Keywords:** serpin (serine proteinase inhibitor), antithrombin (AT), protein Z-dependent protease inhibitor (ZPI), plasminogen activator inhibitor 1 (PAI-1), therapy, protease nexin I (PN-1)

## Abstract

Bleeding and thrombotic disorders result from imbalances in coagulation or fibrinolysis, respectively. Inhibitors from the serine protease inhibitor (serpin) family have a key role in regulating these physiological events, and thus stand out as potential therapeutic targets for modulating fibrin clot formation or dismantling. Here, we review the diversity of serpin-targeting strategies in the area of hemostasis, and detail the suggested use of modified serpins and serpin inhibitors (ranging from small-molecule drugs to antibodies) to treat or prevent bleeding or thrombosis.

## Introduction

**Figure 1:**
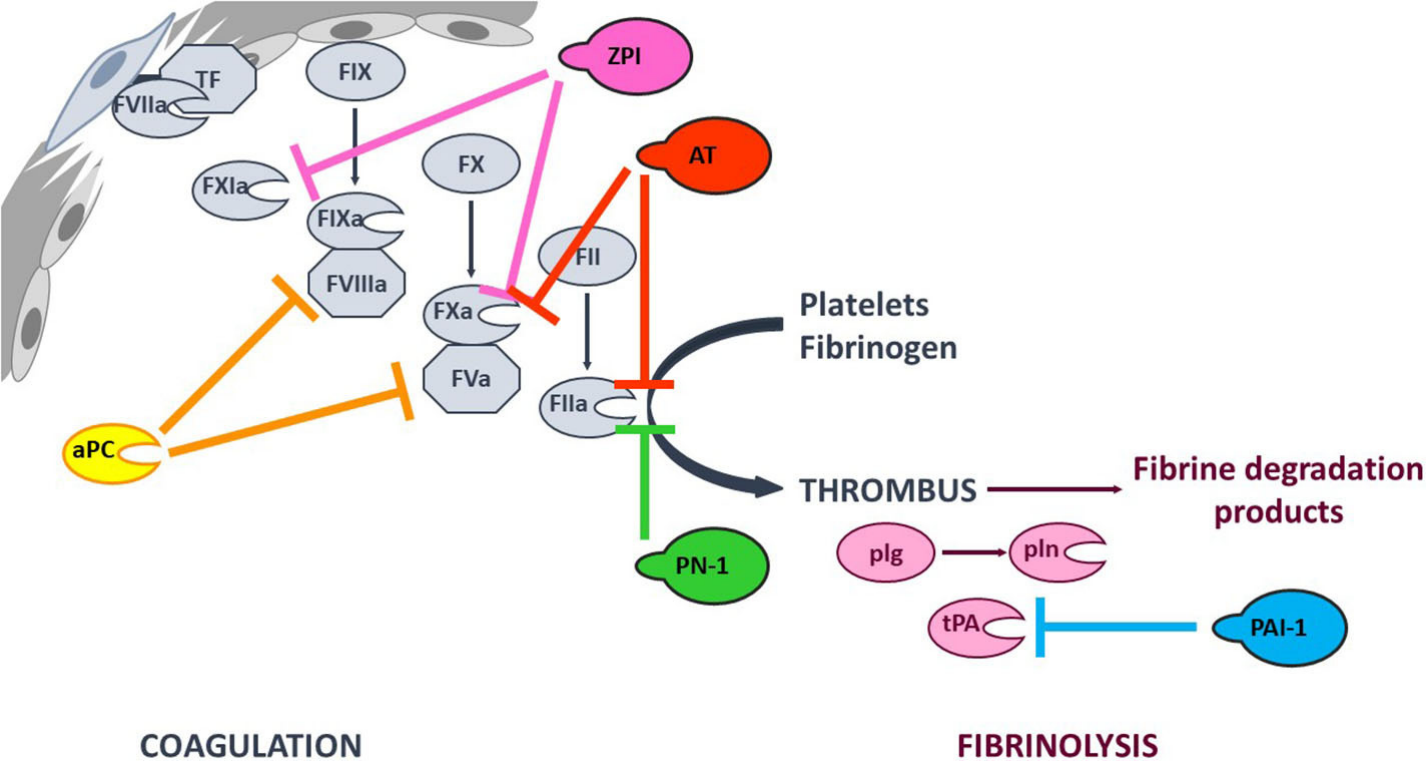
Simplified overview of the coagulation and fibrinolysis processes. Coagulation is initiated at site of injury by TF-FVIIa complex that activates FIX and FX. FXa catalyzes thrombin (FIIa) formation, that amplifies its own generation by activating FXI and cofactors FVIII and FV. Thrombin also promotes platelet aggregation and fibrinogen into fibrin conversion to form a stable thrombus. Fibrinolysis is triggered once the clot is formed by tPA-induced plasminogen (plg) activation. The resulting plasmin (pln) enzyme is then responsible of fibrin degradation. The targets of the principal anticoagulant and antifibrinolytic serpins, as well as that of anticoagulant aPC are indicated with color capped arrows.

Coagulation (the formation of a solid fibrin clot at the site of vessel injury, to stop bleeding) and fibrinolysis (the disaggregation of a clot, to prevent the obstruction of blood flow) are interconnected pathways that both help to maintain the hemostatic balance. Coagulation is initiated at site of injury by exposure of tissue factor (TF), which forms a complex with circulating factor VIIa (FVIIa) and thus activates FIX and FX. In turn, FXa catalyzes the conversion of prothrombin into thrombin—the last enzyme in the coagulation pathway. Thrombin amplifies its own generation by activating cofactors FVIII and FV, which bind to their cognate enzymes (FIXa and FXa, respectively) - thereby considerably increasing their reactivity. Furthermore, thrombin promotes platelet aggregation and fibrinogen proteolysis – leading to the formation of a firm thrombus composed of polymerized fibrin and platelet aggregates (1). Fibrinolysis is triggered once the clot is formed, since fibrin acts as an essential cofactor for plasminogen activation and plasmin-catalyzed fibrin degradation (2). Since coagulation and fibrinolysis proceed by amplifying cascades of enzymatic reactions, these pathways must be finely tuned to prevent excessive bleeding or thrombosis. This fine tuning is performed by (i) cofactors that potentiate or modulate enzyme reactivity, and (ii) protease inhibitors (Figure 1).

Coagulation and fibrinolysis are predominantly regulated by protease inhibitors from the serpin superfamily (3). Antithrombin (AT, *SERPINC1*) is one of the most potent physiological anticoagulants by targeting most of the procoagulant enzymes (especially FXa and thrombin). Other serpins are also involved in regulation of the coagulation cascade, such as heparin cofactor II (*SERPIND1*), protein Z-dependent protease inhibitor (ZPI, *SERPINA10*), protease nexin I (PN-1, *SERPINE2*), and protein C inhibitor (*SERPINA5*) (4–8). The fibrinolysis process is also regulated by serpins. Plasminogen activator inhibitor 1 (PAI-1, *SERPINE1*) inhibits tissue plasminogen activator (tPA) and α2-antiplasmin (*SERPINF2*) inhibits plasmin (9). Members of the serpin family are characterized by a common core structure consisting of 3 β-sheet, 7–9 α-helices, and a variably exposed reactive center loop (RCL). In their native conformation, serpins are in a thermodynamically metastable state and tend to adopt a more stable conformation through insertion of the RCL as a supplementary strand into β-sheet A. This conformational transition occurs either spontaneously (the latent conformation) or upon proteolytic cleavage in the RCL (the cleaved conformation) and serves as the mechanistic basis of protease inhibition by serpins (10). Hence, the serpin-protease reaction follows a branched pathway suicide substrate inhibition mechanism, involving recognition of the RCL as a substrate to form a reversible Michaelis complex. The P1-P1′ scissile bond [according to the nomenclature of Schechter and Berger (11)] is then cleaved, giving an acyl-enzyme intermediate in which the RCL's P1 residue is covalently bound to the protease's catalytic triad. At this point, the two competing branches of the pathway are either (i) hydrolysis of the acyl-enzyme intermediate to yield a cleaved serpin and free proteinase, or (ii) inhibition of the target protease *via* a well-described “mousetrap” mechanism (12). Indeed, upon cleavage, the serpin undergoes a rapid conformational change in which the RCL's N-terminal side inserts into β-sheet A, dragging the protease toward the opposite side of the serpin. Hence, this process traps the protease in an inactive covalent complex with the serpin. Even though a serpin's specificity is mainly determined by the RCL's sequence and its complementarity with the cognate protease's catalytic groove, it also involves exosite interactions between the two protein partners (13). Furthermore, serpin reactivity can be modulated by cofactors that boost the inhibition rate through an allosteric and/or template-based mechanism (14, 15).

Due to the pivotal role of serpins in the regulation of hemostasis and their particular mechanism of action, these proteins have been considered as promising therapeutic targets in the treatment of bleeding or thrombotic disorders (16, 17). Here, we aim at illustrating, through different examples, the broad range of novel strategies used to modulate the activity of serpins involved in coagulation and fibrinolysis. This review focuses principally on basic and preclinical research that might open up novel perspectives and meet unmet needs in the therapeutic management of hemostatic dysfunction.

## Part I: Modified Antithrombin as a Reversal Agent for Heparin Derivatives

**Figure 2:**
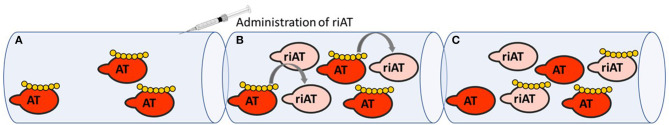
Mechanism of action of riAT as reversal agent for heparin derivatives anticoagulant activity. **(A)** In situation of bleeding risk associated with the use of heparin derivatives (yellow), **(B)** administration of riAT (light pink) could displace heparin derivatives from endogenous AT (red) to riAT by a competition mechanism. **(C)** Hence, heparins are found trapped in an inactive complex with riAT, reducing thereby their apparent anticoagulant activity.

Any discussion of serpins as therapeutic targets must necessarily cover the therapeutic use of heparins (18). Historically, heparin constituted the first class of drug that acted by modulating the reactivity of anticoagulant serpins. Thanks to knowledge of how heparin derivatives bind to antithrombin, how antithrombin is conformationally activated and how procoagulant enzymes are inhibited, the clinical development of heparin derivatives has continued—notably with the release of low-molecular-weight heparin and fondaparinux (the minimal pentasaccharide sequence required to potentiate AT anticoagulant activity) (19–21). When fondaparinux was approved for clinical use, it presented several advantages over unfractionated and low-molecular-weight heparins. It was safer because of its synthetic origin and its favorable pharmacokinetic profile, and it was at least as effective as heparins in several indications (22). However, fondaparinux's main shortcoming is the lack of an effective reversal agent to neutralize its anticoagulant activity (23). Protamine is the only agent approved for heparin-derivative reversal but it is ineffective for fondaparinux and can lead to adverse events (24, 25). Faced with the unmet need for a safe reversal agent for all heparin derivatives, our group engineered a recombinant inactive AT (riAT) that was devoid of anticoagulant activity but retained the ability to bind tightly to heparin. This was achieved by inserting a proline between arginine 393 and serine 394 residues within the RCL and by replacing asparagine 135 with a glutamine residue. The combination of these two mutations dramatically reduced the rate of FXa and thrombin inhibition (by about a 1000-fold) and significant enhanced (by about 3-fold) the AT's affinity for fondaparinux. This riAT was subsequently shown to act as specific, efficacious antidote to heparin derivatives by competing with native AT in plasma (Figure 2) (26). In *ex vivo* studies, riAT dose-dependently reversed anticoagulant activity of fondaparinux, unfractionated heparin, and low-molecular-weight heparin, and was found to be as effective as protamine in neutralizing heparin in a thrombin generation assay. Furthermore, the riAT did not exhibit any apparent prothrombotic activity in the latter assay (27). In *in vivo* models, the intravenous administration of riAT to mice treated with high-dose fondaparinux considerably reduced the fondaparinux plasma concentration (as assessed in a chromogenic assay) (26). Lastly, riAT has also been studied for its ability to neutralize the anticoagulant effects of heparin after a cardiopulmonary bypass (CPB) procedure. The results obtained in a rat model of CPB showed that (in contrast to protamine) riAT was not associated with adverse events (such as hemodynamic instability and histamine release) but was as potent as protamine in reversing heparin's anticoagulant activity. These properties suggested that riAT could safely replace protamine in a post-CPB anticoagulant/reversal protocol (28). Although riAT is promising, its immunogenicity and safety must be evaluated before it can be considered for therapeutic use in humans.

## Part II: Serpin-Targeting Strategies in the Treatment of Hemophilia

Hemophilia is a bleeding disorder that results from a deficiency in FVIII (hemophilia A) or FIX (hemophilia B). Replacement therapy *via* the intravenous infusion of the missing factor is the current standard of care for hemophilia, although it requires frequent administrations and can induce the development of alloantibodies (inhibitors). The limitations of standard treatments have prompted a search for other therapeutic options that correct the bleeding phenotype by downregulating natural anticoagulants and thereby rebalancing hemostasis. This type of approach would be suitable for patients with hemophilia A or B and with or without inhibitors. In this respect, the inhibition of anticoagulant serpins (notably AT) and the use of modified serpins that promote a procoagulant response appear to be promising strategies (Figure 1) (17, 29).

### Inhibition of at in the Treatment of Hemophilia

The most advanced strategy in this field is currently in late-stage clinical development. It consists in silencing AT expression using a small interfering RNA (siRNA, fitusiran). The results of a Phase I clinical trial demonstrated a reduction in the AT level of more than 75% from baseline, together with enhanced thrombin generation in patients receiving the siRNA. In a Phase II trial, a reduction in the AT level of ~80% was observed; this resulted to a fall in the median annualized bleeding rate to 1 in all patients, 48% of whom were bleed-free and 67% of whom did not experience any spontaneous bleeding episodes. Although fitusiran has shown promise, this siRNA might inherently limited by its mechanism of action, which induces inertia of action upon treatment initiation and discontinuation (30, 31). Accordingly, Barbon et al. developed an alternative strategy by engineering a bivalent single-domain antibody (sd-Ab) against AT, which binds to AT and abrogates its anticoagulant activity. The bivalent sd-Ab successfully restored thrombin generation in plasma and reduced blood loss in a FVIII-deficient mouse model of tail vein transection. Furthermore, long-term efficiency and safety of this bivalent sd-Ab in hemophilic mice have been demonstrated following gene transfer with an adeno-associated virus vector. Several weeks after vector administration, the sd-Ab was present in the plasma of treated mice and retained its ability to rescue the bleeding phenotype. No signs of a prothrombotic state or an immune response were evidenced. The strength of this approach lies in the sd-Ab's direct mechanism of action and the short half-life of unbound sd-Ab- warranting its consideration as an alternative strategy in the management of hemophilia (32).

### Use of a Modified Serpin Targeting Activated Protein C in the Treatment of Hemophilia

Another strategy involves targeting activated protein C (aPC). In association with its cofactor protein S, aPC catalyzes the rapid degradation of the cofactors FVa and FVIIIa and thereby shuts down prothrombinase and tenase complex activity (33). Thus, inhibition of aPC might prolong thrombin propagation and restore the hemostatic balance in patients with hemophilia. The challenge was thus to design a modified serpin that potently inhibits aPC but cross-reacts minimally with procoagulant enzymes. After a structural analysis revealed that the catalytic groove of aPC is more permissive to bulky and basic residues than that of thrombin, Polderdijk et al. built a modified serpin on the scaffold of the Pittsburgh variant of a1-antitrypsin, in which the native P2-P1′ sequence Pro-Arg-Ser was replaced by the Lys-Arg-Lys sequence. This modified serpin failed to inhibit thrombin and conserved its strong inhibition of aPC, despite residual inhibitory activity against FXa and FXIa. Importantly, the modified serpin dose-dependently promoted thrombin generation in the presence of thrombomodulin in a thrombin generation assay and restored defective procoagulant activity in FIX-deficient mouse models of bleeding and thrombosis (34). The safety and efficacy of this modified serpin is currently being evaluated in a Phase I/II clinical trial in healthy volunteers and in patients with severe hemophilia (35).

### Protease Nexin-1 and Protein Z-Dependent Protease Inhibitor as Alternative Targets in the Treatment of Hemophilia

Although inhibition of AT or aPC appears to a promising strategy for the treatment of hemophilia, other anticoagulant serpins, such as PN-1 and ZPI have also been investigated in this respect. PN-1 is stored within α-granules in platelets and is released from activated platelets during the clotting process, where it acts as a potent inhibitor of thrombin and FXIa (7). ZPI is a plasma protein that inhibits two key procoagulant enzymes, FXa and FXIa. Whereas, FXIa is rapidly inhibited by free ZPI, FXa inhibition requires the presence of protein Z as a cofactor for ZPI (6). The anticoagulant properties of PN-1 and PZ/ZPI system have been evidenced in a mouse model of FeCl_3_-induced thrombosis, in which deletion of the gene coding for PN-1, ZPI, or PZ significantly increased vessel occlusion (7, 36). The relevance of counterbalancing the bleeding phenotype in hemophilia by blocking the PN-1 or PZ/ZPI system was highlighted by work on double-knockout mouse models. When combined with FVIII deficiency, PN-1 gene deletion tended to limit tail bleeding after vein or artery transection (37). Similarly, PZ or ZPI gene deletion in FVIII-deficient mice attenuated bleeding in a tail vein rebleeding model (38). It was also shown that polyclonal antibodies against PN-1 or PZ (to neutralize ZPI-mediated FXa inhibition) were able to promote thrombin generation in plasma from patients with hemophilia (37, 38). These findings have prompted the search for PN-1 and ZPI inhibitors with potential therapeutic value. A modified ZPI bearing two alanine mutations (the first in the RCL's P1 position, precluding protease inhibition, and the second in the PZ binding site, enhancing the affinity for PZ by about 20-fold) have been designed to serve as a decoy for PZ. By competing with native ZPI for PZ, the modified ZPI was found to antagonize PZ/ZPI anticoagulant activity and promote thrombin generation in plasma from patients with hemophilia (39). With a similar objective, bivalent sd-Abs directed against PN-1 have also been described recently. These sd-Abs specifically bind to and inhibit PN-1, so that they successfully neutralize PN-1-induced elongation of the clotting time in normal or FVIII-deficient plasma (40). However, it remains to be determined whether the procoagulant response produced by neutralization of PN-1 or ZPI is sufficient to prevent bleeding, since to date, none of these strategies has proved its efficacy *in vivo*. Nevertheless, these targets might offer safer alternatives with regard to the risk of thrombotic adverse events; this emerged as a potential issue when a Phase II clinical trial of fitusiran had to be temporarily interrupted because of a case of fatal thrombosis (31). Although AT and PC deficiencies are undoubtedly associated with an increased risk of thrombosis, this link is much less conspicuous with ZPI and PN-1 deficiencies. In contrast to AT or PC, whose gene deletion results in perinatal or embryonic lethality, ZPI or PN-1 knockout mice are viable (7, 36, 41, 42). In addition, while clinical studies clearly evidence an increased risk of thrombosis in patients with AT or PC deficiency, they lead to conflicting results regarding the role of ZPI and fail to establish a convincing association between ZPI deficiency and thrombophilia (43, 44).

## Part III: Targeting Plasminogen Activator Inhibitor Type-1 in the Treatment of Thrombosis

The serpin PAI-1 is also involved in hemostasis because it is the main regulator of fibrinolysis. PAI-1's antifibrinolytic properties (due to the inhibition of tPA) have prompted a search for PAI-1 inhibitors of value in the treatment of thrombotic disorders. In the vasculature, tPA is considered to be the main promoter of fibrinolysis; it binds to fibrin and converts plasminogen into plasmin, which in turn catalyzes fibrin proteolysis (45). Along with the use of anticoagulant drugs to downregulate the procoagulant pathway, treatment of thrombosis may require the use of thrombolytic therapy to restore blood flow in occluded vessels. To this end, tPA infusion has been successfully used as a thrombolytic agent in the management of acute myocardial infarction, ischemic stroke, and pulmonary embolism. However, it is also associated with life-threatening adverse bleeding events (46). Given that PAI-1 deficiency is characterized only by mild-to-moderate bleeding without spontaneous bleeding episodes (whereas high plasma levels of PAI-1 have been linked to the development of thrombosis), PAI-1 inhibition might safely promote thrombolysis in the treatment of vascular thrombosis (45). Several research groups have attempted to develop small-molecule PAI-1 inhibitors (47, 48). The most intensively studied PAI-1 inhibitor to date is tiplaxtinin (also referred to as tiplasinin or PAI-039). This compound blocks the formation of the covalent complex between PAI-1 and its target proteases, and thus converts PAI-1 into a substrate. Thanks to docking studies and mutagenesis experiments, researchers have determined that the tiplaxtinin binding site is in the helix D/E region—close to PAI-1's vitronectin binding site. The results of *in vivo* studies in rats and dogs have shown that tiplaxtinin is a highly potent accelerator of thrombus re-permeabilization after acute carotid injury (49). Other molecules with anti-PAI-1 activity have been described; for example, our group identified annonacinone (a natural product found in plants of the Annonaceae family) via high-throughput screening. Like tiplaxtinin, annonacinone binds to an hydrophobic pocket between the β-sheet stand 2A and the α-helixes D and E (close to PAI-1's vitronectin binding site) and inhibits PAI-1/tPA complex formation by enhancing PAI-1's behavior as a substrate. Annonacinone is more potent than tiplaxtinin in inhibiting PAI-1 *in vitro*, with IC_50_ values of 9 and 28 μM, respectively, in a chromogenic assay. Annonacinone promotes fibrinolysis in plasma (as assessed by thromboelastography) and potentiates tPA-induced thrombolysis in a murine model of FeCl_3_-induced venule occlusion (50). However, the compound's highly challenging chemical synthesis and lack of efficacy against vitronectin-bound PAI-1 have not warranted the clinical development of annonacinone. Another family of chemical compounds as PAI-1 inhibitors was developed through virtual screening and structure-activity relationship studies. In particular, the molecule TM5275 which specifically inhibits PAI-1, presents the advantage to be orally bioavailable. Docking simulations suggested that TM5275 binds to strand 4 of the PAI-1's β-sheet A. Like tiplaxtinin and annonacinone, TM5275 inhibits PAI-1 activity *in vitro* by favoring its substrate behavior. Orally administered TM5275 exhibits antithrombotic activity in rodent and non-human primate models of thrombosis and does not significantly lengthen the bleeding time (51). However, in spite of these promising results, no clinical development of TM5275 has been reported yet. Finally, although several studies have suggested that small molecule PAI-1 inhibitors could provide benefits in the treatment of thrombotic events, whether used alone or in combination with tPA, none of them have entered clinical development as thrombolytic agents. Because in plasma, PAI-1 is predominantly bound to vitronectin that stabilizes its active conformation, one possible explanation is that most of these molecules inhibit PAI-1 less effectively when the latter is bound to vitronectin because of overlapping binding sites between inhibitors and vitronectin (52). In addition, PAI-1 is involved in multiple other functions than fibrinolysis, and PAI-1 inhibitors may exhibit toxicity or adverse drug reaction. Hence, specific regulation of PAI-1 antifibrinolytic activity is an intensively pursued but as-yet unattained objective.

## Conclusion

The studies presented in this review highlight the great interest in serpin-targeting strategies for future therapeutic application in the field of hemostasis. These strategies cover a broad range of approaches to regulate serpins, these include (i) silencing of serpin synthesis, (ii) inhibition of serpin activity by chemical compounds or sd-Abs, and (iii) bioengineering of modified serpin to refine its function or to serve as inactive bait for cofactors. Although the principal aim of hemostasis is to prevent exsanguination after injury, it also participates in the process of wound healing (53). Hence evaluation of the long-term effects of the serpin-targeting strategies on healing beyond their direct impact on coagulation and fibrinolysis will require special attention. The ongoing clinical trials for fitusiran and modified serpin as aPC inhibitor will provide valuable information on efficacy and safety of serpin-targeting strategies for the treatment of hemophilia and will surely motivate further basic and preclinical researches to continuously improve therapeutic management of bleeding and thrombotic disorders.

## Author Contributions

EB performed literature searches and wrote the manuscript. CA, MR, MV, and DB reviewed and edited the review. All authors contributed to the article and approved the submitted version.

## Conflict of Interest

The authors declare that the research was conducted in the absence of any commercial or financial relationships that could be construed as a potential conflict of interest.
